# Characterization of Monoclonal Antibodies Recognizing Citrulline-Modified Residues

**DOI:** 10.3389/fimmu.2022.849779

**Published:** 2022-03-11

**Authors:** Yaqiong Chen, Lin Weng, Wei Liu, Chenxi Deng, Jinxiu Xuan, Yuan Ma, Cao Li, Jinlu Jiang, Juan Chen, Shengxiang Ge

**Affiliations:** ^1^ Department of Rheumatology, The First Affiliated Hospital of Xiamen University, School of Medicine, Xiamen University, Xiamen, China; ^2^ State Key Laboratory of Molecular Vaccinology and Molecular Diagnostics, National Institute of Diagnostics and Vaccine Development in Infectious Disease, School of Public Health, Xiamen University, Xiamen, China

**Keywords:** citrullination, citrulline residues, monoclonal antibodies, high sensitivity, high specificity

## Abstract

**Background:**

Citrullination is a post-translational protein modification linked to the occurrence and development of a variety of diseases. The detection of citrullinated proteins is predominately based on antibody detection although currently available reagents demonstrate detection bias according to the environmental context of the citrullinated residues. This study aimed to develop improved antibody reagents capable of detecting citrullinated residues in proteins in an unbiased manner.

**Methods:**

BALB/c mice were sequentially immunized using citrulline conjugates with different carrier proteins, and specific monoclonal antibodies (mAbs) identified by primary screening using citrulline-conjugated proteins unrelated to the immunogen. Secondary screening was performed to identify mAbs whose reactivity could be specifically blocked by free citrulline, followed by identification and performance assessment.

**Results:**

Two mAbs, 22F1 and 30G2, specifically recognizing a single citrulline residue were screened from 22 mAbs reacting with citrulline conjugates. Compared with commercially available anti-citrulline antibodies (AB6464, AB100932 and MABN328), 22F1 and 30G2 demonstrated significantly higher reactivity as well as a broader detection spectrum against different citrullinated proteins. 22F1 and 30G2 also had higher specificity than commercial antibodies and overall better applicability to a range of different immunoassays.

**Conclusion:**

Two mAbs specifically recognizing a single citrulline residue were successfully produced, each possessing good specificity against different citrullinated proteins. The improved utility of these reagents is expected to make a strong contribution to protein citrullination-related research.

## Introduction

First reported by Rogers and Symonds in 1958 (1), citrullination refers to post-translational modification (PTM) of proteins involving arginine residues where the guanidine group is converted to a carbamido group. This process is catalytically driven by protein arginine deiminases or PADs (2), resulting in the loss of positive charge and two potential hydrogen donors (3). Citrullination makes important contributions to different physiological processes, for instance, citrullination is required for the differentiation of oligodendrocytes and during myelinogenesis, and has also been correlated with skin keratinization (4, 5). Abnormal protein citrullination has also been related to the occurrence and development of multiple disease states. For example, autoantibodies produced due to protein citrullination can be detected in 75% of patients with rheumatoid arthritis (6, 7), and higher antibody titers are associated with worsened patient symptoms (8). Moreover, the extracellular trap formed is one of the sources of autoantigens in patients with systemic lupus erythematosus (9), one of which is citrullinated histones (10). In addition, citrullinated proteins have also been observed in different diseases including Parkinson’s disease (11), Alzheimer’s disease (12), atherosclerosis (13) and cancer (14). Therefore, methods to detect protein citrullination are of great importance for exploring the role of this PTM in different disease states and are essential in the development of new diagnostic markers.

Currently, the analysis of protein citrullination primarily relies on detection using antibody-based (15, 16) and probe-based assays (7, 17, 18) along with mass spectrometry (19, 20). Antibody assays rely on anti-citrullinated protein antibodies to detect citrullinated moieties while, in the probe assay, the sample is pretreated to chemically modify the citrulline residues which are then detected through the analysis of the coupled chemical group (7, 17, 18) or *via* chemical group-modified citrulline residues (15). For mass spectrometry, pre-enrichment of the sample for citrullinated proteins is often needed, which relies on either use of anti-citrulline antibodies or through chemical modification of the samples followed by enrichment with ligands similar to the probe recognition methods. Among these approaches, the anti-citrullinated protein antibodies are the most widely used for different applications including liquid fluid sample detection (21), Western blotting (22), immunofluorescence (11, 23), immunohistochemistry (24, 25) and immunological enrichment (26). However, most of the antibodies used are polyclonal antibodies (27) or monoclonal antibodies (mAbs) (28) obtained by immunization with citrullinated-protein conjugates. However, the epitopes recognized by these antibodies can suffer from recognition bias caused by amino acid residues surrounding the citrullinated residues or other the environmental effects. Therefore, with the currently available reagents, it can be difficult to fully assess the citrullination of different proteins in the sample.

The present study aimed to overcome this limitation by developing improved antibody reagents recognizing citrullinated-proteins. Mice were sequentially immunized using citrullinated peptides with different motifs and after screening antibody-producing clones, two mAbs recognizing free L-citrulline were obtained. These mAbs showed better sensitivity and broad-spectrum recognition properties in the detection of citrullinated proteins than commercially available anti-citrullinated protein antibodies. These mAbs are expected to promote the development of more rigorous methods to detect citrullinated proteins and advance functional studies of protein citrullination under physiological and pathological conditions.

## Materials and Methods

### Animal Care

BALB/c mice aged 6-8 weeks old were purchased from Shanghai SLAC Laboratory Animal Co., Ltd., The animals were housed in individual ventilated cages (IVCs) and maintained in a 12 h light-dark cycle, at a temperature of 22-25C and a relative humidity of 45- 55%, and allowed free access to food and water. All animal experiments were carried out in compliance with the regulations of the Animal Welfare and Ethics Committee at Xiamen University **(**KY2017-026**)**. Animal suffering was minimized or prevented at all times to improve their welfare. And all procedures and animal care were approved in accordance with the *Regulations for the Administration of Laboratory Animals of the People’s Republic of China*.

### Preparation of mAbs

The citrulline-coupled proteins XC-keyhole limpet hemocyanin (KLH), XXXC-KLH, XC-ovalbumin (OVA), XXXC-OVA and XC- Bovine albumin (BSA), and the arginine-coupled protein RC-BSA were synthesized by Sangon Bioengineering (Shanghai) Co., Ltd. (X: citrulline; C: cysteine; R: arginine). All other chemicals were standard commercial products of analytical-reagent grade.

A total of 75 µg of citrulline-coupled protein was mixed with an equal volume of aluminum adjuvant and then injected subcutaneously to immunize mice at 1-week intervals in the order of XC-KLH, XC-OVA, XXXC-KLH and XXXC-OVA. The immunization schedule details were as follows: XC-KLH on day 0, XC-OVA on day 1, XXXC-KLH on day 14 and XXXC-OVA on day 21. Using XC-BSA as the detection antigen, we measured serum antibody titers along with reactivity of the monoclonal cell supernatants. With RC-BSA as the control, monoclonal cells that reacted with XC-BSA but did not react with RC-BSA were selected.

### *In Vitro* Protein Citrullination and Biotin Labeling of Antibodies

Protein citrullination: The protein to be citrullinated was prepared into 0.1 µg/µL solution with deamination buffer (20 mM Tris-HCl, pH 8.8, 3 M NaCl, 1 mM EDTA, 10 mM DTT and 5 mM CaCl_2_). Then 25 µL of the protein solution and 10 µL of PAD (Sigma-Aldrich, P1584) were added into 210 µL of deamination buffer and mixed evenly, followed by deamination at 37°C for 15 h. Finally, the reaction was terminated using 5 µL of 20 mM EDTA solution. Protein citrullination was confirmed by MALDI-TOF mass spectrometry.

Biotin labeling of antibodies: Sulfo-NHS-LC-Biotin (21335) was purchased from ThermoFisher Scientific and used to label the antibody according to the manufacturers’ instructions. The antibody/biotin ratio was 1:10, and the final concentration of the antibody was 1 mg/mL after labeling.

### Enzyme-Linked Immunosorbent Assay (ELISA)

Indirect ELISA: The antigen (XC-BSA, XC-KLH, citBSA, cit-protein phosphatase 1B (ppm1b), cit- Cytomegalovirus interleukin 10 (cmvIL10) was diluted to 500 ng/mL with 50 mM carbonate buffer (pH 9.6). A total of 100 µL of diluted antigen was added into each well of a 96-well plate, incubated at 37°C for 2 h and washed once with phosphate buffered saline with Tween-20 (PBST) (20 mM phosphate buffer, pH 7.4, 150 mM NaCl, 0.05% Tween-20). Thereafter, each well was incubated with 200 µL of blocking buffer (20 mM phosphate buffer, 10% newborn bovine serum, 1% casein, and 1% Triton X-100) at 37°C for 2 h. Antibodies to be tested was diluted with blocking buffer to specific concentrations and added to each well (100 µL) for 1 h at 37°C before washing 5 times with PBST, followed by incubation with 100 µL of species appropriate HRP-labeled secondary antibodies (Invitrogen, 31440) at 37°C for 0.5 h. After washing with PBST 5 times, wells were incubated with 100 µL of 3,3’,5,5’-tetramethylbenzidine (TMB) substrate at 37°C for 15 min before terminating the color reaction with 50 µL of 2 M H_2_SO_4_. Optical density (OD) measurements were conducted at a wavelength of 450 nm with 630 nm used as the reference wavelength. To detect the reaction of antibodies with free citrulline or arginine, the L-citrulline solution or L-arginine [Macklin (Shanghai, China)] solution (pH 7.2) at different concentrations was mixed with the antibody to be tested diluted with an equal volume of blocking buffer, and incubated at 37°C for 1 h. Then 100 µL of the mixture was added into the XC-BSA-coated microplate, and detected in the manner as for the indirect ELISA.

Sandwich ELISA: The anti-citrullinated protein antibody, 22F1 and 30G2 was diluted to 1 µg/mL with 20 mM phosphate buffer (pH 7.2). Next, 100 µL of diluted antibody was added into each well of a 96-well plate, incubated at 37°C for 2 h and washed once with PBST. Then it was incubated with 100 µL of blocking buffer in each well at 37°C for 2 h. The antigen to be tested was diluted with blocking buffer to different concentrations and 100 µL added into each well for 1 h at 37°C before washing 5 times with PBST. Then 100 µL of biotin-labeled anti-citrullinated protein antibody diluted with blocking buffer was added into each well, reacted at 37°C for 1 h, washed with PBST 5 times and reacted with 100 µL of SA-HRP (OR03L, diluted with blocking buffer) at 37°C for 30 min. After washing 5 times with PBST, TMB substrate was added and measured as described above for the indirect ELISA.

### Indirect Immunofluorescence Assay

BSA and citrullinated BSA (citBSA) were diluted with 20 mM phosphate buffer (pH 7.4) to different concentrations. Protein solutions (150 µL) were then applied to nitrocellulose membranes using the Bio-Dot^®^ Microfiltration System (Bio-Rad). After drying, the membrane was blocked with 5% skim milk at room temperature for 2 h, washed once with deionized water, reacted with anti-citrulline antibody (Abcam, AB6464, AB100932 and Millipore, MABN328, diluted with skim milk to 1 μg/mL) at room temperature for 1 h. After washing with PBST 5 times, Alexa Fluor^®^ 488-labeled secondary antibody (Invitrogen, A-21042, diluted with skim milk to 1 µg/mL) was added for incubation at room temperature for 1 h. After washing again with PBST 5 times, the fluorescence intensity of the membrane was observed using the ChemiDoc™ MP Imaging System (Bio-Rad).

### Western Blotting

BSA and citBSA protein samples (2 µg) and PageRuler Plus Prestained Protein Ladder (molecular weight marker) were resolved using 10% sodium dodecyl sulfate-polyacrylamide gel electrophoresis (SDS-PAGE) and transferred onto nitrocellulose membranes. The membranes were then blocked with 5% skim milk at room temperature for 2 h, washed once with deionized water, and reacted with anti-citrulline antibody, include 22F1,30G2, and MABN328 (Millipore) (diluted in skim milk solution to 1 µg/mL) at room temperature for 1 h. After washing with PBST 5 times, HRP-labeled secondary antibody (Invitrogen, 31440, diluted in skim milk solution to 1 µg/mL) was added for incubation at room temperature for 1 h. After washing again with PBST 5 times, WesternBright Sirius chemiluminescent detection kits were used to reveal the immunoreactive bands and images developed using the ChemiDoc™ MP Imaging System.

## Results

### Preparation and Identification of mAbs Against Citrulline Residues

**Figure 1:**
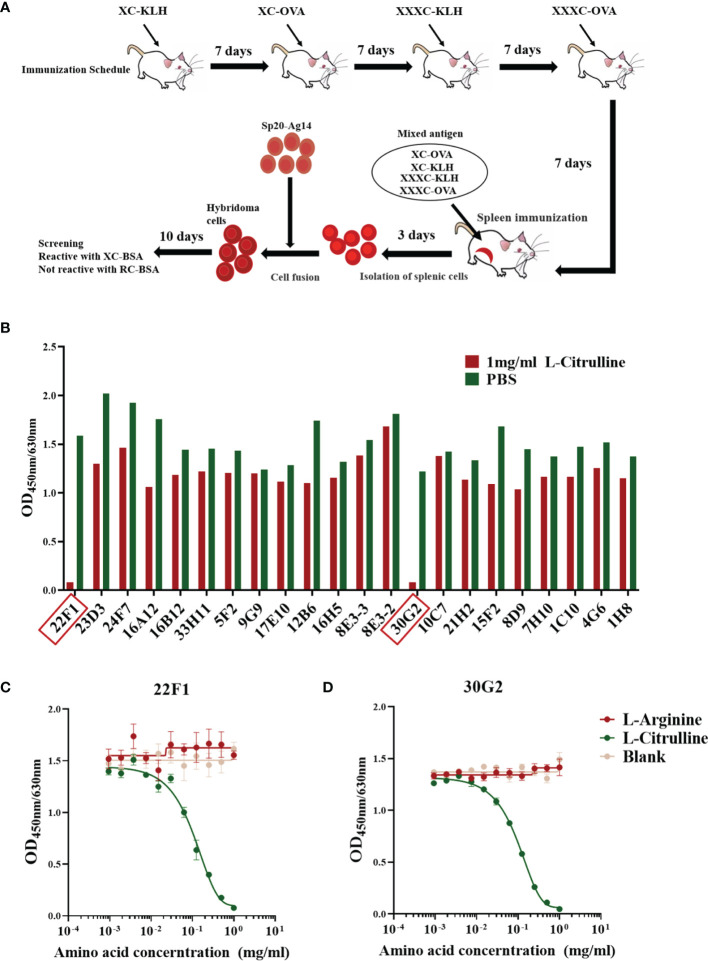
Preparation and identification of mAbs against citrulline residues. **(A)** Immunization and mAb screening. X, citrulline; A, arginine; C, cysteine; KLH, keyhole limpet hemocyanin; OVA, ovalbumin; BSA, bovine serum albumin. **(B)** Reactivity of mAbs with L-citrulline. mAbs 22F1 and 30G2 reactive with L-citrulline are indicated by red boxes. **(C)** Reactivity curve of 22F1 with L-citrulline and L-arginine. **(D)** Reactivity curve of 30G2 with L-citrulline and L-arginine.

The BALB/c mouse immunization scheme and screening process for identifying mAbs is shown in **Figure 1A**. The initial screen using ELISA-based assays identified a total of 22 candidate mAbs that reacted with XC-BSA but not RC-BSA. To further screen for mAbs that could react with free L-citrulline, each mAb was diluted with blocking buffer until the intensity of the reaction against XC-BSA reached an OD value of 1.0-2.0. Then the ability of exogenous L-citrulline to block the binding between each mAbs and XC-BSA was determined using the method described in Section 2.4.

Out of the 22 mAbs screened, the reactivity of only two could be effectively inhibited by free L-citrulline (**Figure 1B**). Specifically, the reactivity of mAbs 22F1 and 30G2 against XC-BSA declined by 90% in the presence of 1 mg/mL L-citrulline. Further assessment showed the inhibitory effects of L-citrulline on the reactivity of mAbs was dose-dependent (**Figures 1C, D**) with the half maximal inhibitory concentration (IC_50_) of L-citrulline against 22F1 and 30G2 being 0.12 mg/mL and 0.10 mg/mL, respectively (**Supplementary Figures 1A, C**). In contrast, the addition of 1 mg/mL L-arginine had no inhibitory effects on the binding activity of 22F1 and 30G2 (**Figures 1C, D** and **Supplementary Figures 1B, D**). According to the identification of antibody subtypes, both 22F1 and 30G2 were of the IgG1 subtype (**Supplementary Figure 2**).

### Reactivity of 22F1 and 30G2 With Citrullinated Proteins

To verify the reactivity of 22F1 and 30G2 against citrullinated proteins, we prepared *in vitro* citrullinated forms of BSA, ppm1b and cmvIL10 and citrulline conjugates (XC-BSA and XC-KLH). In addition, we used the commercial anti-citrulline antibodies AB6464, AB100932 and MABN328 as controls.

**Figure 2:**
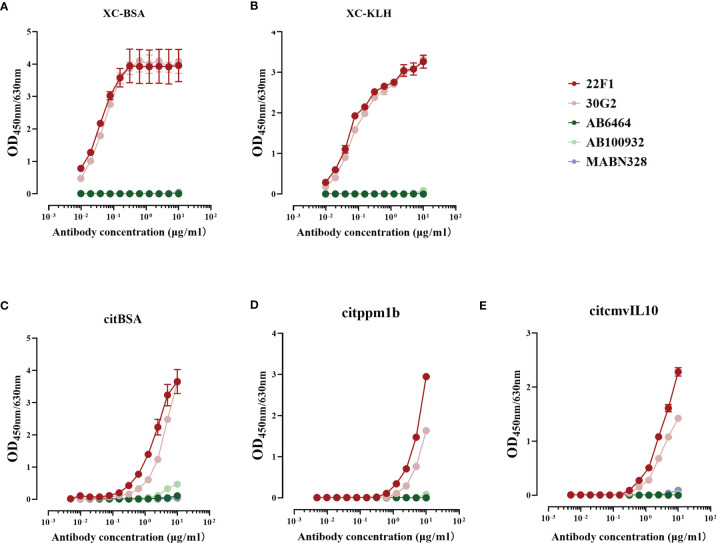
Reactivity of five antibodies with citrulline conjugates and citrullinated proteins. **(A)** XC-BSA, **(B)** XC-KLH. **(C)** citBSA. **(D)** citppm1b. **(E)** citcmvIL10. Citrulline conjugates, XC-BSA and XC-KLH; citrullinated proteins, citBSA, citppm1b and citcmvIL10. X, citrulline; A, arginine; C, cysteine; KLH, keyhole limpet hemocyanin; BSA, bovine serum albumin; ppm1b, protein phosphatase 1B; cmvIL10, cytomegalovirus interleukin 10.

Our results revealed that the reactivity of 22F1 and 30G2 with citrullinated proteins or conjugates was markedly higher than either of the three commercial anti-citrullinated protein antibodies. Consistent with the screening data, 22F1 and 30G2 showed good reactivity against XC-BSA and XC-KLH while the three commercial antibodies showed negligible reactivity (**Figures 2A, B**). Similarly, 22F1 and 30G2 detected XC-KLH, citBSA and citppm1b but none of the three commercial antibodies produced significant reactivity except for AB100932 at high concentrations against citBSA (**Figures 2C–E**). Importantly, 22F1 and 30G2 did not react with non-citrullinated proteins such as BSA, KLH, ppm1b and cmvIL10 even at concentrations of 10 µg/mL, indicating good specificity (**Supplementary Figure 3**). The three commercial antibodies at a concentration of 10 µg/mL did not react with BSA, KLH and ppm1b, but AB100932 and MABN328 showed some cross-reactivity with cmvIL10 (**Supplementary Figure 3**). Considering that negative reactivity was typically below OD values of 0.010, we set a cut-off OD value of 0.100 to analyze the lowest concentration of antibody that reacts with the antigen (**Table 1**). Using this approach, it was found that the reactivity of 22F1 was slightly higher than that of 30G2, but both reagents had good reactivity with different antigens containing citrulline residues, and their reactivity was at least 10 times higher than that of commercial antibodies.

**Table 1:** The minimum concentration of anti-citrullinated protein antibodies that react with different antigens.

Antigens	Antibodies (µg/mL)				
	22F1	30G2	AB6464	AB100932	MABN328
Citrulline conjugates and citrullinated proteins					
XC-BSA	<0.010^a^	<0.010	>10^b^	>10	>10
XC-KLH	<0.010	<0.010	>10	10	>10
citBSA	0.078	0.156	10	2.5	>10
citppm1b	0.625	1.25	>10	10	>10
citcmvIL10	0.313	0.625	>10	>10	10
Non-citrullinated proteins					
BSA	>10	>10	>10	>10	>10
KLH	>10	>10	>10	>10	>10
ppm1b	>10	>10	>10	>10	>10
cmvIL10	>10	>10	10	0.078	>10

a if a given antibody at the lowest concentration of serial dilutions has reactivity above OD 0.100, its minimum reactive concentration is defined as less than the lowest concentration.

b if a given antibody at the highest concentration of serial dilutions has reactivity below OD 0.100, its minimum reactive concentration is defined as more than the highest concentration.

### Detection of Citrullinated Protein by Double-Antibody Sandwich Immunoassay

**Figure 3:**
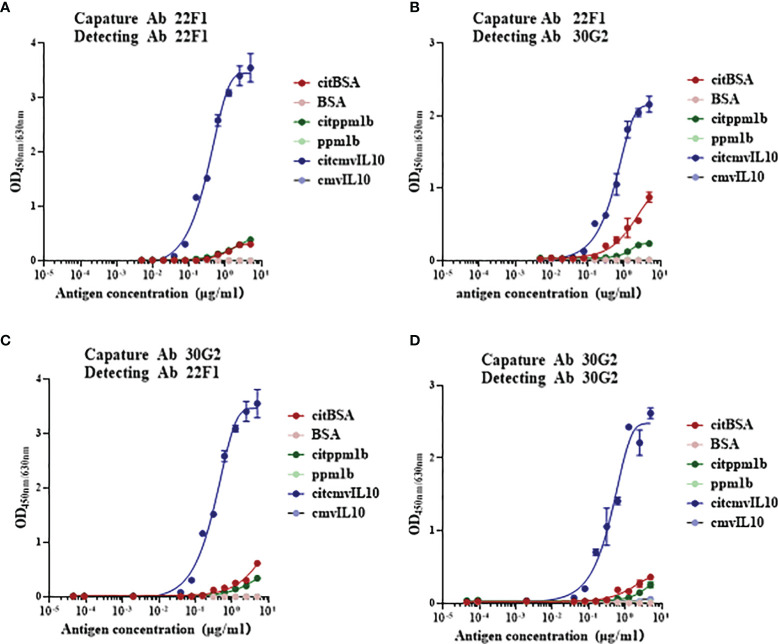
Reactivity of citrullinated proteins detected by citrulline residue specific antibodies based on sandwich immunoassays. **(A)** Antibody pair of 22F1 and 22F1, **(B)** Antibody pair of 22F1 and 30G2, **(C)** Antibody pair of 30G2 and 22F1, and **(D)** Antibody pair of 30G2 and 30G2. The citrullinated proteins, such as citBSA, citppm1b and citcmvIL10, were detected, while their non-citrullinated proteins were used as controls. BSA: bovine serum albumin.

Since there is often more than one citrullinated arginine residue in citrullinated proteins, we constructed double-antibody sandwich immunoassays to determine if the combination of 22F1 and 30G2 mAbs could improve the detection of multi-citrullinated proteins. It was found that self-paired or mutually-paired 22F1 and 30G2 could successfully detect citrullinated proteins such as citBSA, citppm1b and citcmvIL10 (**Figure 3**). No significant difference was observed among the four pairs, and the lower limit of detection was between 10 ng/mL and 500 ng/mL. The reactivity of citcmvIL10 was significantly higher than that of citBSA and citppm1b. Assays with non-citrullinated BSA, ppm1b and cmvIL10 demonstrated no significant reactivity at concentrations of 5,000 ng/mL.

### Reactivity of 22F1 and 30G2 With citBSA in Indirect Immunofluorescence and Western Blotting Assays

**Figure 4:**
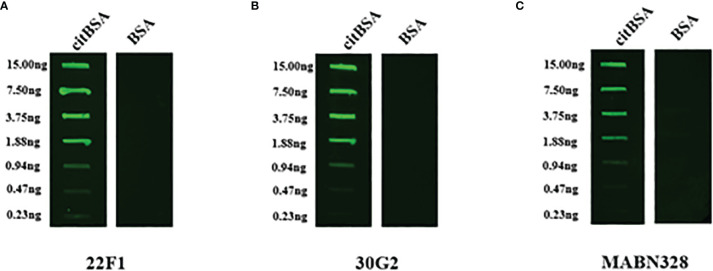
Indirect immunofluorescence assay. citBSA and BSA were two-fold diluted from an initial concentration of 100 ng/mL to a final concentration of 1.57 ng/mL, and 150 μL of each dilution was spotted onto a nitrocellulose membrane. The primary antibodies used were **(A)** 22F1, **(B)** 30G2 and **(C)** MABN328. BSA: bovine serum albumin; ppm1b: protein phosphatase 1B; cmvIL10, cytomegalovirus interleukin 10.

**Figure 5:**
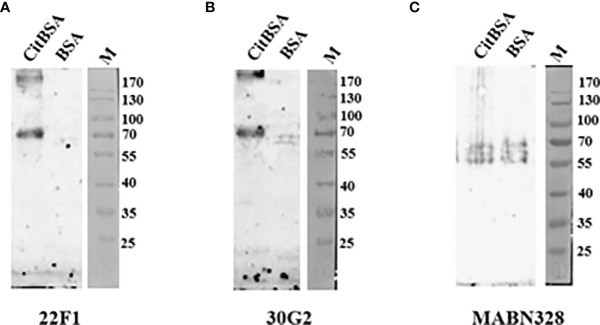
Western blotting assay. 2 μg of each citBSA and BSA was loaded and analyzed, and Western blotting was performed with different primary antibodies. **(A)** Western blotting result of 22F1, **(B)** Western blotting result of 30G2, **(C)** Western blotting result of MABN328. M, molecular weight marker. BSA, bovine serum albumin.

Next, we further explored the utility of 22F1 and 30G2 in other types of immunoassays, testing their reactivity with citBSA in indirect immunofluorescence and western blotting assays. As a control we included MABN328 as this showed the best specificity among the three commercial antibodies. In the indirect immunofluorescence assays, the reactivity of 22F1 with citBSA was slightly higher than that of 30G2, consistent with the result of the indirect ELISA. Although MABN328 almost had no reactivity with citBSA in the indirect ELISA, its reactivity with citBSA was only slightly lower than that of 30G2 in the indirect immunofluorescence assay (**Figure 4**). In the Western blotting assay, both 22F1 and 30G2 decorated a specific band at the expected position of the citBSA protein while there were almost no obvious bands in the BSA lane. In contrast, MABN328 reacted with multiple bands in both citBSA and BSA lanes (**Figure 5**), indicating no specificity towards citrullinated residues in BSA.

## Discussion

Protein citrullination not only participates in the occurrence and development of rheumatoid arthritis (29), but also involves a variety of other types of inflammatory diseases (30). In order to explore the role of protein citrullination in the occurrence and development of these diseases, it is of great importance to better understand which proteins are citrullinated and also, to what extent. Citrullinated proteins are commonly characterized by the presence of citrulline residues in their amino acid sequences (31). However, citrulline alone possesses no immunogenicity, so it is often coupled with carrier protein as an immunogen to prepare anti-citrulline antibodies which can be either polyclonal (27) or monoclonal (28) antibodies. The specificity of polyclonal antibodies is maintained through carrier protein adsorption while mAbs are differentially screened for clones that do not react with the carrier protein. Irrespectively, these antibodies recognize epitopes composed of citrulline residues together with adjacent amino acid residues on the carrier protein, and thus cannot consider to directly bind to citrullinated residues alone. Therefore, the specificity of anti-citrulline antibodies depends more or less on their surrounding environmental amino acids. As a result, their abilities to recognize different types of citrullinated proteins can greatly vary, and it is difficult to equate reactivity with the true levels of protein citrullination in the sample. To overcome this limitation, we used to citrulline-couple KLH and OVA for sequential immunization followed by citrulline-coupled BSA for antibody reactivity testing. Screening for positive clones whose reactivity could be blocked by free L-citrulline resulted in the identification of two mAbs, 22F1 and 30G2, that specifically bind to citrulline residues.

In the indirect ELISA, both 22F1 and 30G2 showed good reactivity with different types of citrullinated proteins and citrulline conjugates, including 2 citrullinated proteins (citppm1b and citcmvIL10) not used for mouse immunization and antibody screening. The latter suggests that 22F1 and 30G2 have good broad-spectrum reactivity against different citrullinated proteins, and moreover, infers their binding to citrullinated residues is not influenced by environmental amino acids. In addition to indirect ELISA, 22F1 and 30G2 also displayed good reactivity with citrullinated proteins in indirect immunofluorescence and Western blotting assays, suggesting that they can be used on a variety of immunoassay platforms. In particular, the indirect immunofluorescence assay favors maintaining native protein conformations while in the Western blotting assay, the protein antigens bound to the nitrocellulose membranes are present in a denatured state (32). Both 22F1 and 30G2 showed good reactivity with citrullinated proteins in both assays, indicating that their reactivity with citrullinated proteins is not strongly affected by protein conformation. Importantly, in the indirect ELISA, indirect immunofluorescence, and Western blotting assays, 22F1 and 30G2 did not react with non-citrullinated proteins or carrier proteins not coupled with citrulline, suggesting that both 22F1 and 30G2 possess good specificity.

As an important part of our study, we compared the new mAbs against three widely-used commercial anti-citrullinated protein antibodies (24, 25, 33). In the indirect ELISA, the reactivity of the three commercial antibodies with citrullinated proteins or citrulline conjugates was lower than that of 22F1 and 30G2, and the 2 polyclonal antibodies (AB6464 and AB100932) showed some non-specific reactions with non-citrullinated proteins. In particular, the reactivity of AB100932 with the non-citrullinated protein cmvIL10 was far higher than that with citrullinated protein citcmvIL10. It is speculated that the epitope recognized by AB100932 on cmvIL10 is destroyed after protein citrullination, so its reactivity with citcmvIL10 significantly declines. Strangely, the reactivity of the commercial mAb MABN328 with the citrullinated protein citBSA had obvious differences in different immunoassay methods. Specifically, the reactivity of MABN328 with citBSA was far lower than that of 22F1 and 30G2 in the indirect ELISA, while it was comparable to 30G2 in the indirect immunofluorescence assay, but it almost had no reactivity with citBSA in the Western blotting assay. Moreover, MABN328 had no obvious non-specific reaction with the non-citrullinated BSA in indirect ELISA and indirect immunofluorescence assay, but obvious non-specific bands were found in the Western blotting assay. The likely reason is that the citBSA epitope recognized by MABN328 is conformationally dependent, and sufficiently altered to lose reactivity when citBSA is coated onto polystyrene microplates or subjected to denaturation in Western blotting. The reason for the non-specific bands of MABN328 in the Western blotting assay may be that the epitope able to bind to MABN328 is exposed after SDS-PAGE.

There should be more than one arginine residue on the surface of most proteins, so multiple citrulline residues and multiple sites that can bind to citrulline residue-specific antibodies may also exist on the surface of citrullinated proteins. In view of this, a double-antibody sandwich ELISA system was constructed using 22F1 and 30G2 and used to detect citrullinated proteins. The results showed that different citrullinated proteins could be successfully detected by this method. This system is expected to be used to detect the total amount of citrullinated protein in samples, thereby reflecting the total burden of protein citrullination in samples, and also used to explore the correlation between the total amount of citrullinated proteins and the occurrence and development of diseases.

One potential limitation of our reagents is the fact the reactivity of 22F1 and 30G2 can be blocked by free L-citrulline. Free citrulline is generally produced by the urea cycle (34) or the nitric oxide synthesis (35) and potentially, free citrulline present in biological samples could interfere with the detection of citrullinated proteins. Nevertheless, interference by free citrulline can be readily eliminated by pretreating samples to remove small molecules using techniques such as dialysis and ultrafiltration. Moreover, in some assays, the presence of free citrulline is unlikely to be problematic since methods such as SDS-PAGE and Western blotting inherently separate small molecules from the citrullinated protein analytes which thereby eliminates the influence of free citrulline in the detection steps. In addition interference with free citrulline would probably be avoided by using the antibodies in sandwich assay format, as unlikely to both bind same small epitope simultaneously.

In conclusion, two mAbs specifically recognizing citrulline residues have been successfully prepared in this study, which have better broad-spectrum responses to citrullinated proteins, and higher reactivity and specificity than commercial antibodies. They are applicable to different immunoassay platforms and are expected to significantly advance research related to protein citrullination.

## Data Availability Statement

The original contributions presented in the study are included in the article/**Supplementary Material**. Further inquiries can be directed to the corresponding authors.

## Ethics Statement

The animal study was reviewed and approved by the Animal Welfare and Ethics Committee at Xiamen University (KY2017-026).

## Author Contributions

JC and SG conceived the study. JC acquired funding. YC and LW collected the data, conducted data analysis, and drafted manuscript. YC, LW, WL, CD, JX, YM, CL, and JJ performed laboratory tests. JC and SG were responsible for supervision of the study. All authors critically reviewed the manuscript and approved the final version.

## Funding

This study was funded by the National Natural Science Foundation of China (Grant No. 81771751) to JC and Fujian Provincial Department of Science and Technology (Grant No. 2018J01383) to JC.

## Conflict of Interest

The authors declare that the research was conducted in the absence of any commercial or financial relationships that could be construed as a potential conflict of interest.

## Publisher’s Note

All claims expressed in this article are solely those of the authors and do not necessarily represent those of their affiliated organizations, or those of the publisher, the editors and the reviewers. Any product that may be evaluated in this article, or claim that may be made by its manufacturer, is not guaranteed or endorsed by the publisher.
