# Supplementary material for: Characterization of Monoclonal Antibodies Recognizing Citrulline-Modified Residues
Source: Front Immunol. 2022 Mar 11;13:849779. doi: 10.3389/fimmu.2022.849779 (PMC8961739; doi:10.3389/fimmu.2022.849779)
Supplement: Supplementary file 1 [file DataSheet_1.pdf]

Supplementary Figure 1

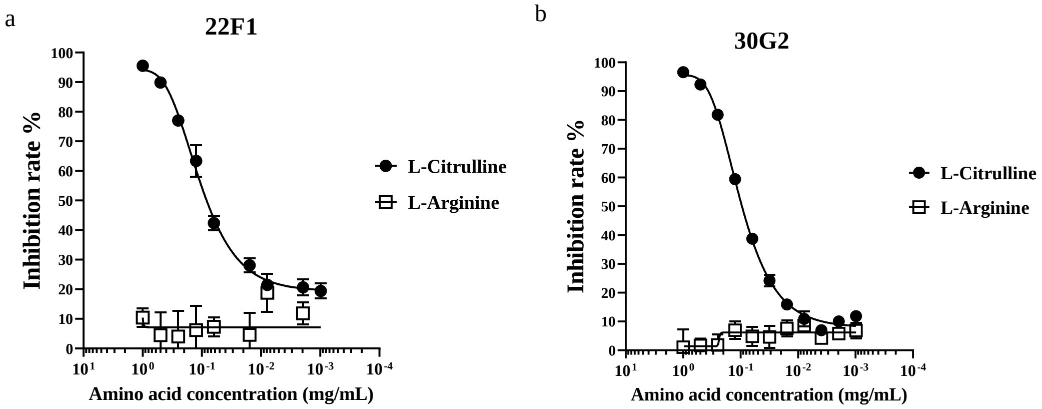

Supplementary Fig. 1 The inhibition of L-citrulline and L-arginine on the reactivity of monoclonal antibodies with XC-BSA. a. 22F1 and **b.** 30G2. The solutions of L-citrulline and L-arginine were adjusted to pH 7.2, and then diluted with 20 mM, pH 7.2 phosphate buffer to indicated concentration. The inhibition rate was calculated by the formula: [1 – (mean OD values of a given concentration of free amino acid / mean OD values of absence of free amino acid)] * 100%. The reactivity of 22F1 and 30G2 with XC-BSA can be inhibited by L-citrulline with IC50 of 0.12 mg/ mL and 0.10 mg/ mL respectively, while the reactivity cannot be suppressed by L-arginine.

Supplementary Figure 2

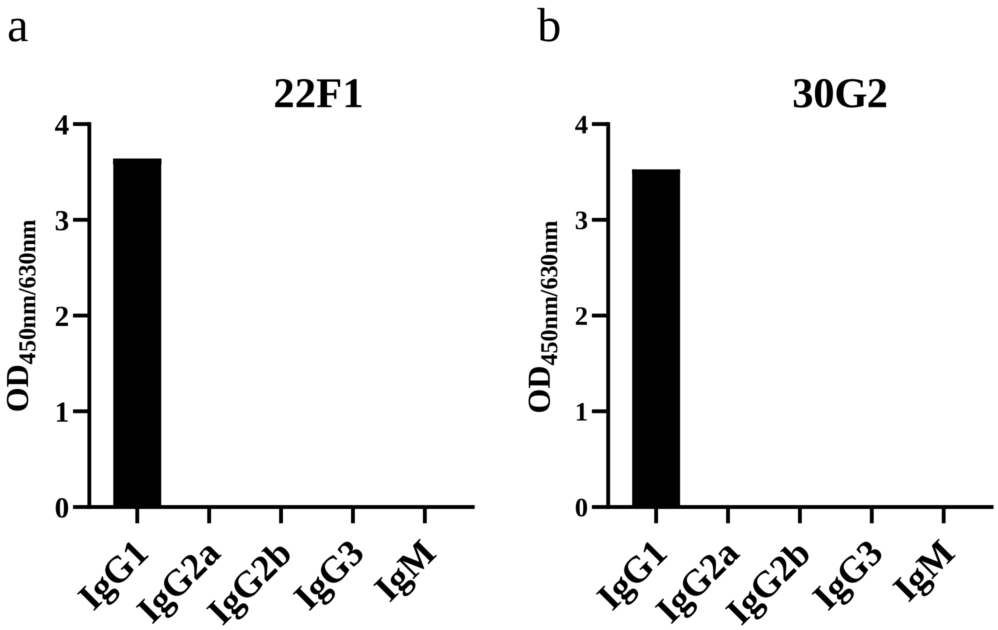

Supplementary Fig. 2 Isotyping of 22F1 and 30G2. Mouse Immunoglobulin Isotyping ELISA Kit was purchased from BD biosciences (550487). The results indicated that both 22F1 and 30G2 are subtype of IgG1.

Supplementary Figure 3

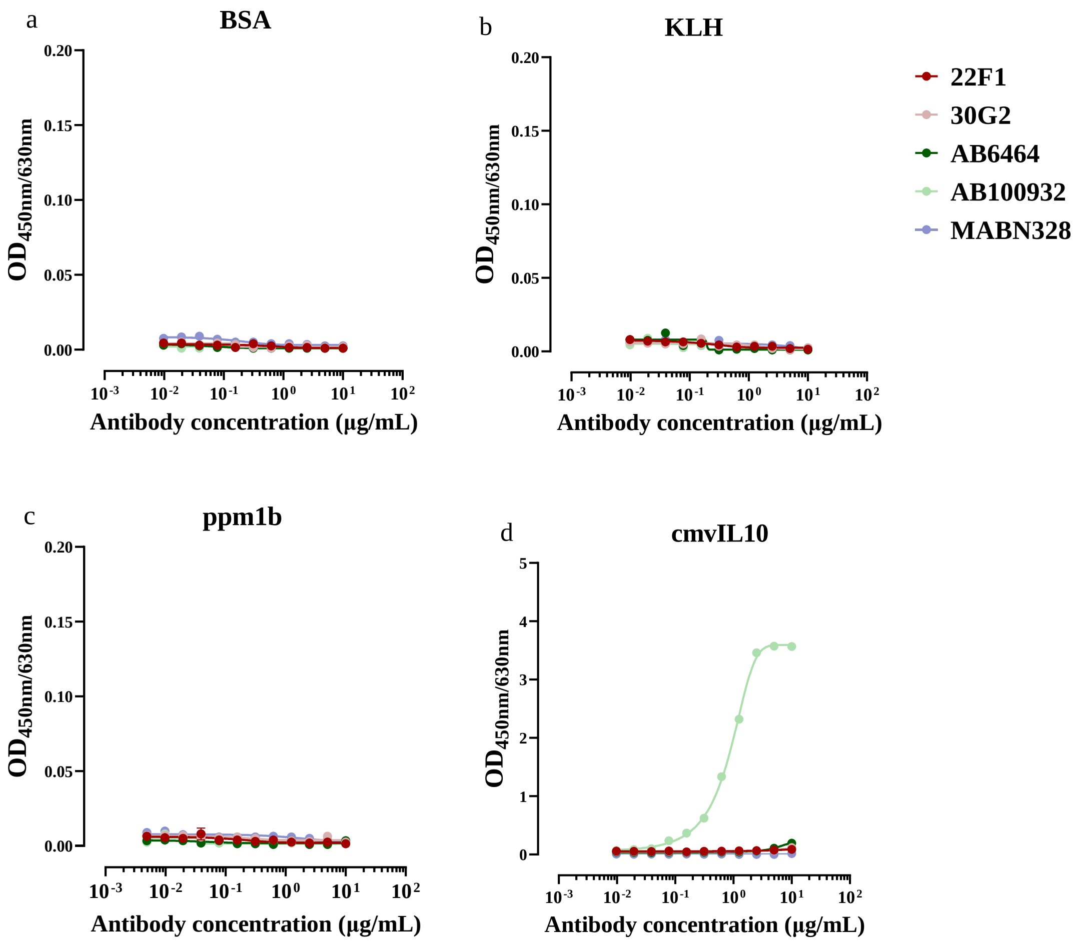

Supplementary Fig. 3 Reactivity of different antibodies with uncitrullinated proteins. a. BSA, **b.** KLH, **c.** ppm1b and **d.** cmvIL10.
